# Circular RNA circ_0001287 inhibits the proliferation, metastasis, and radiosensitivity of non-small cell lung cancer cells by sponging microRNA miR-21 and up-regulating phosphatase and tensin homolog expression

**DOI:** 10.1080/21655979.2021.1872191

**Published:** 2021-01-20

**Authors:** Chuan-Cui Zhang, Yuhua Li, Xian-Zhen Feng, Dian-Bo Li

**Affiliations:** a Department of Respiratory, The Third People’s Hospital of Linyi, Linyi, China;; b Department of Oncology, The Third People’s Hospital of Linyi, Linyi, China; c Department of Thoracic Surgery, Linyi Cancer Hospital, Linyi, China

**Keywords:** circ_0001287, miR-21, PTEN, NSCLC

## Abstract

As a type of non-coding RNA, circular RNA (circRNA) figures prominently in human cancer progression. Nonetheless, the expression, function, and regulatory mechanism of circ_0001287 in non-small cell lung cancer (NSCLC) remain obscure. In this work, quantitative real-time polymerase chain reaction (qRT-PCR) was implemented to quantify circ_0001287 and miR-21 expressions in NSCLC tissues and cells. The relationship between circ_0001287 expression and the clinicopathological parameters of NSCLC patients was examined. Cell counting kit-8 (CCK-8), 5-bromo-2©-deoxyuridine (BrdU), and Transwell experiments were conducted to detect the multiplication, migration, and invasion of NSCLC cells after circ_0001287 was overexpressed or knocked down. The survival of NSCLC cells was studied using colony formation experiment under different doses of radiation. RNA immunoprecipitation (RIP) experiment and luciferase reporter gene experiment verified the binding relationship between circ_0001287 and miR-21. Western blot was employed to examine the regulatory effects of circ_0001287 and miR-21 on phosphatase and tensin homolog (PTEN) expression. We reported that circ_0001287 expression was down-modulated in NSCLC tissues and cell lines. Besides, circ_0001287 low expression was associated with low differentiation and positive lymph node invasion of NSCLC. Circ_0001287 overexpression suppressed the multiplication, migration, invasion, and radioresistance of NSCLC cells, whereas circ_0001287 knockdown promoted the above phenotypes. Circ_0001287 could adsorb miR-21 and repress its expression, and indirectly up-modulate PTEN expression in NSCLC cells. Taken together, circ_0001287/miR-21/PTEN axis is probably involved in regulating NSCLC cell multiplication, metastasis, and radioresistance.

## GRAPHICAL ABSTRACT

**Figure:**
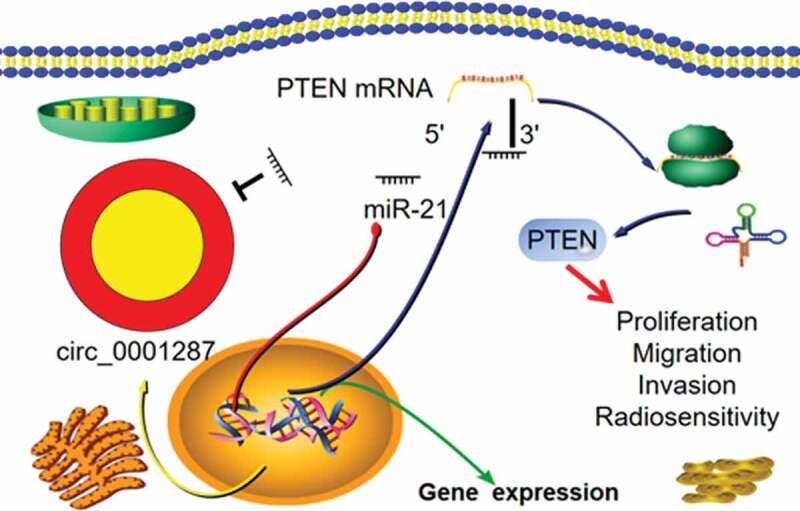

## Introduction

Lung cancer ranks first as the cause accounting for cancer-related deaths worldwide, with non-small cell lung cancer (NSCLC) taking up about 85% of all lung cancer cases [1,2]. The majority of the patients are diagnosed with advanced tumors and their 5-year survival rate is below 20% [3]. Exploring the mechanism of NSCLC progression is of great significance to explore novel therapy strategy.

Circular RNA (circRNA) is a novel endogenous non-coding RNA (ncRNA) characterized by circular structure [4]. Due to this closed-loop structure, to some extent, it helps circRNA resist degradation by RNase, which highlights the advantages of circRNAs as a stable molecular biomarker for diverse cancers [5–7]. circRNAs are also crucial regulators in cancer biology [8–10]. For example, circ-PRMT5 aggravates the malignant characters of breast cancer cells by activating the PI3K/AKT pathway and up-modulating TCF7L2 expression [8]. So far, there are few researches on circ_0001287, and its role in NSCLC has yet to be studied.

MicroRNAs (miRNAs) are ncRNAs with approximately 20–25 nucleotides in length, which modulate gene expression by inhibiting the translation of mRNA or degrading target mRNAs [11–13]. They regulate the pathogenesis of human diseases, including cancers [14–17]. MicroRNA-21 (miR-21) acts as an oncomiR in NSCLC, enhancing cancer cell multiplication, migration, invasion, and radioresistance [18]. Interestingly, CircInteractome database indicates the existence of putative binding sites between circ_0001287 and miR-21 (context score percentile = 99, which shows a high possibility of regulatory relationship between them), implying that circ_0001287 may impede NSCLC progression by working as a miR-21 sponge.

In the present study, we performed *in vitro* experiments and substantiated that circ_0001287 expression was down-modulated in NSCLC and its overexpression repressed the multiplication, metastasis, and radioresistance of NSCLC cells. We also experimentally reasoned that circ_0001287 could negatively and directly modulate miR-21 expression and induce the expression of tumor-suppressor phosphatase and tensin homolog (PTEN). The findings signify that circ_0001287/miR-21/PTEN pathway is a novel mechanism of NSCLC progression, and our work provides clues for NSCLC diagnosis and treatment.

## Materials and methods

### Clinical data

NSCLC tissue specimens (n = 87) and matched normal adjacent tissues were obtained from the patients who received surgery in the Third People’s Hospital of Linyi and instantly stored in liquid nitrogen. The patients signed written informed consent before surgery, and this work was endorsed by the Research Ethics Committee of Linyi Cancer Hospital.

### Cell lines and cell culture

Human NSCLC cell lines (H1299, SPC-A1, A549, H2170, and H157) and normal human bronchial epithelial cell line (16HBE cells) were from the American Type Culture Collection (ATCC, Manassas, VA, USA). All cells were cultured in RPMI-1640 medium (Invitrogen, Carlsbad, CA, USA) containing 10% heat-inactivated fetal bovine serum (FBS, HyClone, Logan, UT, USA), 100 U/mL penicillin, and 100 μg/mL streptomycin (Invitrogen; Thermo Fisher Scientific, Inc., Waltham, MA, USA). The medium was refreshed every 3–4 d. The cells were trypsinized using 0.25% trypsin (HyClone, Logan, UT, USA) for subculture.

### Cell transfection

The circular transcript expression vector had two components called the front circular and the back circular frame, which were designed to contain flanks of inverted repeat sequences. To overexpress circ_0001287, the full-length cDNA of circ_0001287 was amplified and cloned into the specific vector between the two frames. To knock down circ_0001287, according to the circ_0001287 junction sequence, siRNAs were designed to specifically target circ_0001287. The overexpression vector and siRNAs were provided by GenePharma (Shanghai, China). H1299 and A549 cells were collected, and transferred into a 6-well plate (2 × 10^5^ cells/mL), and cultured for 24 h. Then H1299 and A549 cells were transfected using Lipofectamine® 2000 (Invitrogen, Carlsbad, CA, USA) in line with the protocols. Transfection efficiency was detected by quantitative real-time polymerase chain reaction (qRT-PCR).

### qRT-PCR

Total RNA was extracted from tissues and cells using TRIzol® reagent (Invitrogen, Carlsbad, CA, USA). Subsequently, 1 μg of total RNA was reversely transcribed into cDNA using SuperScript First-strand cDNA System (Invitrogen, Carlsbad, CA, USA). The qRT-PCR was then carried out with SYBR Premix Ex Taq (TaKaRa, Dalian, China) on ABI StepOne Plus Real-time PCR system (Applied Biosystems, Foster City, CA, USA). The relative expressions of circ_0001287, miR-21, and PTEN were calculated using 2^−ΔΔCT^ method. The primers were as follows: circ_0001287: 5© - CCAGCAAATCTCCAGTGGTT-3© (forward) and 5©-TGGCAAACTGTTCTTTAGCTTTT-3© (reverse); miR-21: 5©-ACACTCCAGCTGGGTAGCTTATCAGACTGA-3© (forward) and 5©-TGGTGTCGTGGAGTCG-3© (reverse); PTEN: 5©-CAGAAGACTTGAAGGCGTAT-3© (forward) and 5©-AGCAGAGAATGGAAAGTCAAA-3© (reverse); U6: 5©-CTCGCTTCGGCAGCACA-3© (forward) and 5©-AACGCTTCACGAATTTGCGT-3© (reverse); GAPDH: 5©-CACCATCTTCCAGGAGCGAG-3© (forward) and 5©-TCACGCCACAGTTTCCCGGA-3© (reverse).

### Cell counting kit-8 (CCK-8) assay

Cell multiplication was appraised using the CCK-8 kit (Dojindo, Kumamoto, Japan) in compliance with the manufacturer’s instruction. Thereafter, H1299 and A549 cells were transferred into a 96-well plate (1 × 10^3^ cells/well) and cultured for 24 h. Then, 10 μL of CCK-8 kit was supplemented to each well before the cells were incubated for 1 h. The absorbance (OD_450nm_) was detected on a microplate reader (Bio-Rad, Hercules, CA, USA), and similarly, the absorbance of the cells was measured on the 2^nd^, 3^rd^, 4^th^, and 5^th^ day, respectively.

### 5-bromo-2©-deoxyuridine (BrdU) assay

NSCLC cells were prepared into single-cell suspension and planted on slides positioned in 12-well plates (1 × 10^5^ cells/well). After NSCLC cells adhered to the bottom of the wells, 10 μmol/L of BrdU solution was added, and the cells were incubated for 4 h at 37°C. The solution was then discarded and the cells were rinsed 3 times with PBS. Subsequently, 70% ethyl alcohol was added, and the cells were fixed at 4°C for 10 min, and after 70% ethyl alcohol was discarded, the cells were rinsed using PBS 3 times. Then, 2 mol/L of HCl was added and maintained at 37°C for 40 min to denature DNA. Thereafter, HCI was discarded and the cells were rinsed with PBS 3 times. After 1% FBS was supplemented, the cells were blocked for 1 h. Then, the cells were rinsed with PBS again, and anti-BrdU monoclonal antibody (Abcam, ab1893, 1:300) was added to each well, and the cells were incubated overnight at 4°C. On the next day, Cy3 labelled goat anti-mouse fluorescent secondary antibody was added and incubated with cells for 2 h. Ultimately, the nuclei were counterstained with DAPI and the cells were observed under fluorescence microscope. Four fields were randomly taken from each slide, and the number of BrdU-positive cells was counted. Cell multiplication rate = the number of BrdU staining positive cells/total number of cells×100%.

### Transwell assay

In the invasion assay, Matrigel (1:10; BD Biosciences, Franklin Lakes, NJ, USA) was utilized to coat the Transwell chambers (8 μM, BD Biosciences, San Jose, CA, USA) while in the migration assay, Matrigel was not used. Transfected cells were resuspended in serum-free medium and transferred into in Transwell compartment (5 × 10^4^ cells/well), and the medium containing 10% FBS was supplemented in the 24-well plate. After the cells were cultured for 36 h, the cells passing through the membrane were fixed with methanol and stained with crystal violet solution. After that, the number of cells was counted under a light microscope (magnification, x100; Olympus, Tokyo, Japan).

### Colony formation experiment

Cell colony formation experiment was employed to assess the radiosensitivity of cells. NSCLC cells were transferred into 6-well plates (1000 cells/well). After the cells were exposed to different doses of radiation (0, 2, 4, 6, and 8 Gy), the culture was continued for 2 weeks. Then the medium was discarded, and the colonies were fixed with formaldehyde for 10 min and stained with 0.5% crystal violet solution for 10 min. After rinsing three times with PBS, the stained colonies were counted with naked eyes. Colony formation rate = the number of colonies/1000 × 100%.

### Western blot

After the medium was discarded, the cells were lysed with RIPA lysis buffer (Beyotime, Shanghai, China), and the supernatant was gathered after high-speed centrifugation. After quantifying the protein using BCA reagent (Pierce, Rockford, IL, USA), the supernatant was heated in a thermostat water bath at 100°C for 10 min to denature the protein. Thereafter, the total protein (30 µg/lane) underwent SDS-PAGE. Then the protein was transferred to a PVDF membrane (Millipore, Bedford, MA, USA), and blocked in 5% skim milk for 1 h at room temperature. Following that, the membrane was incubated with anti-PTEN antibody (Abcam, ab170941, 1:1000) and anti-GAPDH antibody (Abcam, ab9485, 1:3000) overnight at 4°C. Subsequently, the PVDF membrane was rinsed with TBST solution and incubated with Goat Anti-Rabbit IgG H & L (Abcam, ab125900, 1:1000) for 1 h at room temperature. After the membrane was rinsed with TBST solution again, the protein bands were developed using hypersensitive ECL (Amersham Pharmacia Biotech, Little Chalfont, UK).

### Dual-luciferase reporter gene assay

CircInteractome database was used for predicting the binding site. The sequence containing the binding site between circ_0001287 and miR-21 was amplified and inserted into pGL3 reporter vector (Promega, Madison, WI, USA) to obtain the wide-type plasmid (circ_0001287-WT). The mutant reporter vector (circ_0001287-MUT) was obtained by site-directed mutagenesis. Lipofectamine® 2000 was used to transfect the reporter vectors and miR-21 mimics or mimics control into 293 T cells. After 48 h, the cells were collected, and the luciferase activity was detected using the dual-luciferase reporting kit (Promega, Madison, WI, USA) following the manufacturer’s instruction. The activity of firefly luciferase was normalized by the activity of renilla luciferase.

### RNA immunoprecipitation (RIP) experiment

Magna RIP™ RNA-Binding Protein Immunoprecipitation Kit (Millipore, Shanghai, China) was adopted to conduct RIP experiments. The cells were collected and resuspended in RIP lysis buffer (Beyotime, Shanghai, China), and the cell extract was incubated overnight with RIP buffer containing magnetic beads coupled to anti-Ago2 antibody (Millipore, Shanghai, China) or IgG. Subsequently, after washing 3 times, the magnetic beads were incubated with proteinase K at room temperature. Total RNA in the immunoprecipitation was then extracted using TRIzol reagent. Ultimately, the relative abundance of circ_0001287 and miR-21 was measured by qRT-PCR.

### Statistical analysis

SPSS 17.0 statistical software (SPSS Inc., Chicago, IL, USA) was adopted to process the data. All experiments in this study were repeated at least 3 times independently, and the measurement data were expressed as mean ± standard deviation (x ± s). Multivariate comparisons were analyzed by one-way ANOVA, and independent sample *t*-tests were used for comparing the difference between two groups. Chi-square test was used to determine the correlation between circ_0001287 expression and the clinicopathological parameters of NSCLC patients. *P* < 0.05 signified the statistical significance.

## Results

### The down-regulation of circ_0001287 expression in NSCLC and its clinical implication

**Figure 1.**
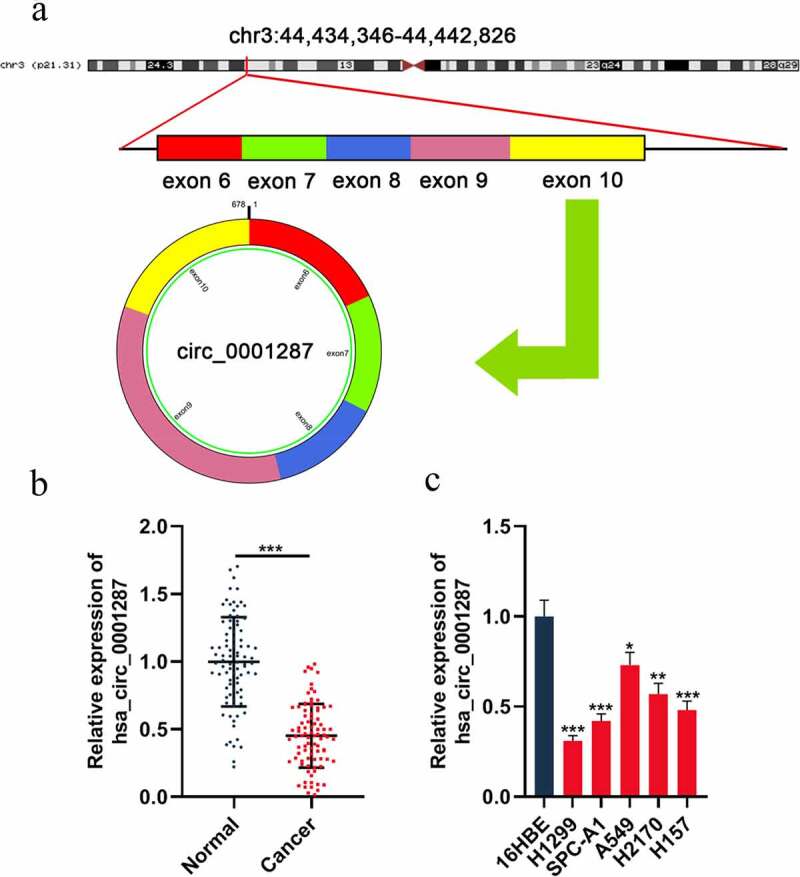
The expression of circ_0001287 in NSCLC

Circ_0001287 is originated from TCAIM gene exon 6–10, with a spliced mature sequence length of 678 base pairs (bp) (Figure 1a). To probe into the circ_0001287 expression in NSCLC, qRT-PCR was executed to examine circ_0001287 expression in cancer tissues and paracancerous tissues of 87 NSCLC patients. The data showed that circ_0001287 was significantly lowly expressed in NSCLC tissues relative to that in paracancerous tissues (Figure 1b). As expected, consistently, circ_0001287 expression in all of the five NSCLC cell lines, compared to in 16HBE cell lines, was reduced (Figure 1c). The association between circ_0001287 expression and clinicopathological parameters in NSCLC patients was further examined. The result elucidated that low circ_0001287 expression was remarkably linked to the positive lymph node metastasis and low differentiation of NSCLC tissues (Table 1). The above data implied that circ_0001287 might exert a tumor-suppressive effect.

**Table 1.** The correlation between hsa_circ_0001287 and clinicopathological features of NSCLC patients

Characteristics	Case number	hsa_circ_0001287 expression		*P* value
		High	Low	
Age				
≥50	52	21	31	0.565
<50	35	12	23	
Gender				
Male	53	21	32	0.685
Female	34	12	22	
Smoking Status				
Smoker	44	14	30	0.235
Non-smoker	43	19	24	
T status				
T1-2	44	21	23	0.057
T3-4	43	12	31	
N status				
N0	34	18	16	0.021*
N1/2	53	15	38	
Histological grade				
Well and moderately	40	20	20	0.032*
Poorly and others	47	13	34	

Note:P-value was determined by Pearson chi-square tests. *P < 0.05.

circ_0001287 is significantly down-regulated in NSCLC tissues and cell lines. *In vitro* assays in this work show that circ_0001287 suppresses NSCLC cell proliferation, migration, invasion, and radioresistance of NSCLC cell. Mechanistically, it is revealed that circ_0001287 can increase PTEN expression via competitively binding with miR-21 in NSCLC cells.

(a) Circ_0001287 was derived from TCAIM gene exon 6–10, with a spliced mature sequence length of 678 bp. (b) qRT-PCR was used to detect the expression level of circ_0001287 in 87 pairs of NSCLC tissues and paracancerous tissues. (c) The expression level of circ_0001287 in NSCLC cell lines (H1299, SPC-A1, A549, H2170, and H157 cells) and normal cell line (16HBE cells) was detected by qRT-PCR. The experiments were repeated three times. * *P* < 0.05, ** *P* < 0.01, and *** *P* < 0.001.

### Circ_0001287 inhibited NSCLC cell multiplication and metastasis, and enhanced its radiosensitivity

**Figure 2.**
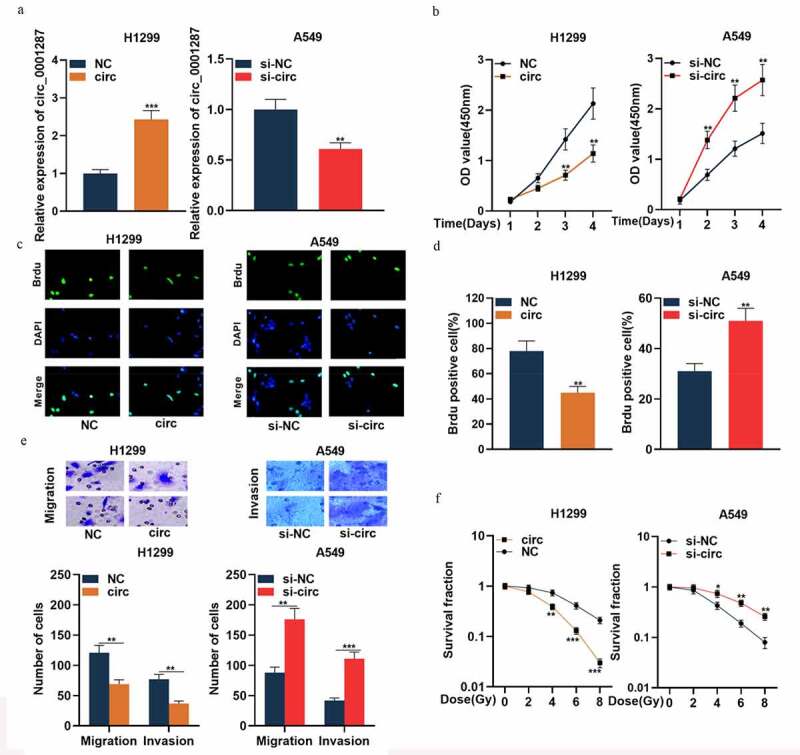
Circ_0001287 inhibited the multiplication, migration, invasion, and radioresistance of NSCLCs

To expound the association between circ_0001287 expression and NSCLC progression, H1299 cells were selected to construct the circ_0001287 overexpression model, and A549 cells were used for the construction of circ_0001287 knockdown model (Figure 2(a)). Following that, functional experiments were performed. The data of the CCK-8 experiment and BrdU assay showed that relative to the control group, circ_0001287 overexpression remarkably suppressed the multiplication of H1299 cells (Figure 2(b-d)). Subsequently, we found through Transwell experiments that circ_0001287 overexpression inhibited H1299 cell migration and invasion (Figure 2(e)). The results of the colony formation experiments suggested that under different doses of radiation, circ_0001287 overexpression significantly inhibited the survival of H1299 cells (Figure 2f). Conversely, relative to the control group, knocking down circ_0001287 expression in A549 cells promoted multiplication, migration, invasion, and radioresistance (Figure 2(b-f)). These data indicated that circ_0001287 was a vital regulator of the malignant phenotypes of NSCLC cells.

(a) The empty plasmid or pcDNA-circ_0001287 plasmid was transfected into H1299 cells, respectively; si-NC or si-circ_0001287 was transfected into A549 cells. Transfection efficiency was verified by qRT-PCR. (b-d) The multiplication of NSCLCs was detected by CCK-8 method and BrdU experiment. (e) Transwell assay was used to detect the migration and invasion of NSCLC cells. (f) Under different doses of radiation, the survival of NSCLC cells was tested by colony formation experiments. The experiments were repeated three times. * *P* < 0.05, ** *P* < 0.01, and *** *P* < 0.001.

### Circ_0001287 adsorbed miR-21 in NSCLC cells

**Figure 3.**
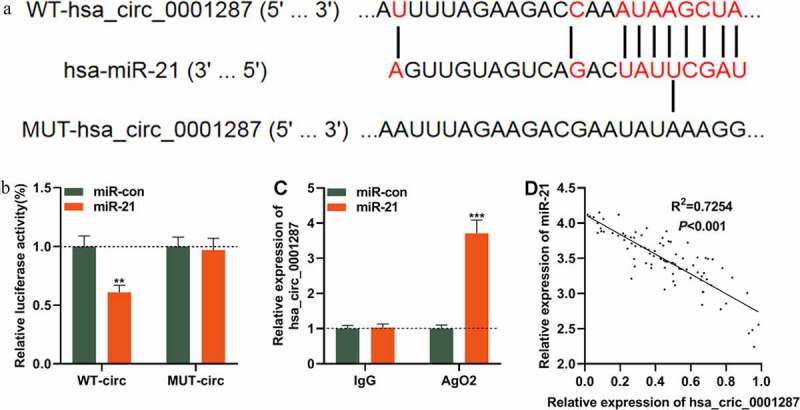
Circ_0001287 adsorbed miR-21

We then tried to determine whether circ_0001287 could act as a miRNA sponge. Interestingly, CircInteractome database predicted that there was a potential binding site between circ_0001287 and miR-21 (Figure 3(a)). To validate whether circ_0001287 could decoy miR-21, dual-luciferase reporter assay was implemented. We found that miR-21 mimics could markedly diminish the luciferase activity of the circ_0001287-WT reporter plasmid while miR-21 mimics had no obvious influence on the luciferase activity of the circ_0001287-MUT reporter plasmid (Figure 3(b)). Thereafter, RIP test manifested that compared with control IgG, circ_0001287 and miR-21 were enriched in Ago2-containing microribonucleoproteins (Figure 3(c)). In addition, Pearson’s correlation analysis demonstrated that miR-21 expression was negatively correlated with circ_0001287 expression in NSCLC samples (R^2^ = 0.7254, Figure 3d). Collectively, it was concluded that circ_0001287 adsorbed miR-21 and negatively regulated its expression in NSCLC cells.

(a) Bioinformatics was used to predict the potential binding site between circ_0001287 and miR-21. (b) Wild type (WT) circ_0001287 or mutant type (MUT) circ_0001287 luciferase reporter vector and miR-con or miR-21 mimics were co-transfected into 293 T cells to measure the luciferase activity of the luciferase reporter vector. (c) RIP experiments confirmed that circ_0001287 and miR-21 were enriched in Ago2-containing microribonucleoproteins. (d) Pearson’s analysis showed that circ_0001287 expression was negatively correlated with miR-21 expression in NSCLC tissues. The experiments were repeated three times. ** *P* < 0.01 and *** *P* < 0.001.

### MiR-21 exerted the cancer-promoting effect in NSCLC

**Figure 4.**
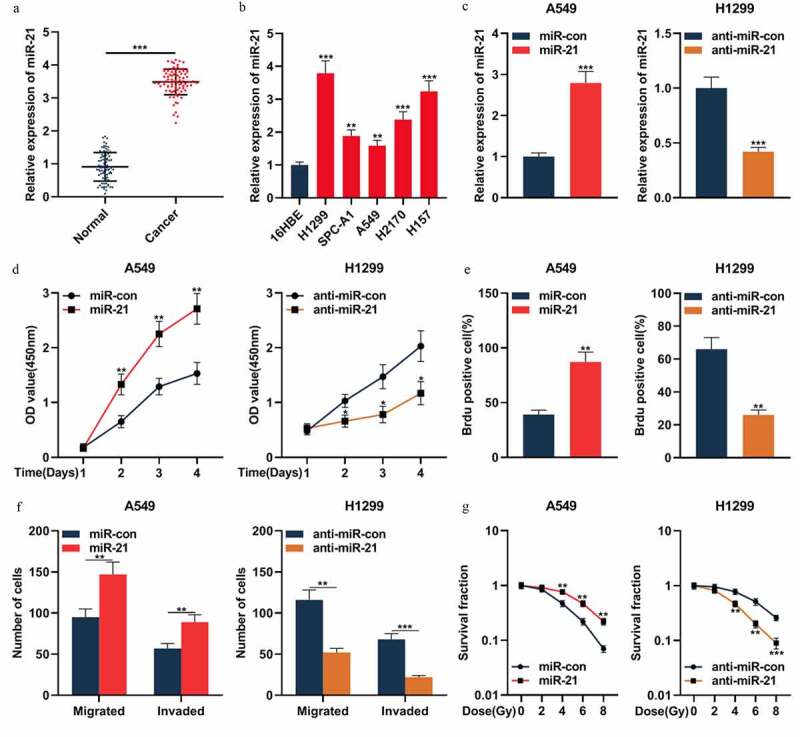
MiR-21 played an oncogenic role in NSCLCs

The above results indicated that miR-21 was one of the downstream targets of circ_0001287, so we then explored the expression characteristics and biological function of miR-21 in NSCLC. qRT-PCR confirmed that miR-21 expression was remarkably up-modulated in NSCLC tissues relative to that in paracancerous tissues (Figure 4(a)). Additionally, miR-21 expression was markedly augmented in all five NSCLC cell lines compared to that in 16HBE cells (Figure 4(b)). To pinpoint the biological effects of miR-21 in NSCLC, miR-21 mimic was transfected into A549 cells, and miR-21 inhibitor was transfected into H1299 cells (Figure 4(c)). Then the multiplication, metastasis, and radiosensitivity of NSCLC cells were appraised by CCK-8, BrdU, Transwell, and colony formation experiments, respectively. The data showed that the up-regulation of miR-21 expression promoted the multiplication, migration, invasion, and radioresistance of A549 cells (Figure 4(d-g)). Conversely, in H1299 cells, suppressing miR-21 expression resulted in the inhibition of the malignant phenotypes of the cancer cells (Figure 4(d-g)). The above data confirmed that miR-21 was oncogenic in NSCLC.

(a) The expression of miR-21 in 87 pairs of NSCLC tissues and paracancerous tissues was detected by qRT-PCR. (b) The expression of miR-21 in NSCLC cell lines (H1299, SPC-A1, A549, H2170, and H157) and normal cell line 16HBE was detected by qRT-PCR. (c) The expression of miR-21 in A549 cells transfected with miR-21 mimics and H1299 cells transfected with miR-21 inhibitors was detected by qRT-PCR. (d–e) CCK-8 method and BrdU experiment were used to detect the multiplication of NSCLC cells. (f) Transwell assay was used to monitor cell migration and invasion of NSCLC cells. (e) Under different doses of radiation, the survival of NSCLC cells was tested by colony formation experiments. The experiments were repeated three times. * *P* < 0.05, ** *P* < 0.01, and *** *P* < 0.001.

### Circ_0001287 mediated malignant biological behaviors of NSCLC cell through the miR-21/PTEN pathway

**Figure 5.**
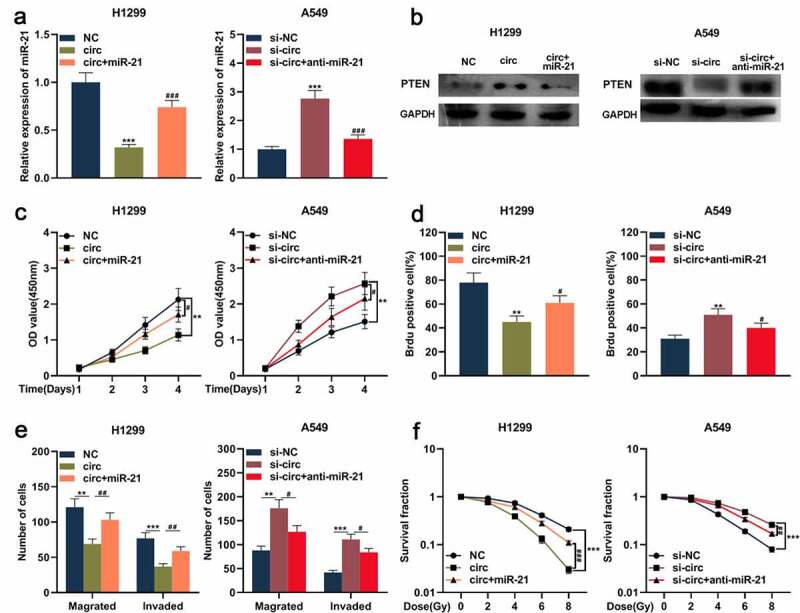
Circ_0001287/miR-21/PTEN axis was involved in regulating the malignant phenotype of NSCLCs

Previous studies report that the miR-21/PTEN axis is involved in regulating NSCLC progression [19–23]. Therefore, we then further explored whether circ_0001287 could indirectly regulate PTEN expression via modulating miR-21 and then affect the development of NSCLC. MiR-21 mimics were transfected into H1299 cells with circ_0001287 overexpression, and miR-21 inhibitors were transfected into A549 cells with circ_0001287 knockdown. qRT-PCR and Western blot showed that circ_0001287 overexpression reduced miR-21 expression, while PTEN protein expression was significantly increased (Figure 5(a-b)). Conversely, miR-21 expression was upregulated after knocking down circ_0001287, while PTEN expression was decreased (Figure 5(a-b)). Moreover, miR-21 mimics or inhibitors partially reversed the regulation of miR-21 and PTEN expressions induced by circ_0001287 (Figure 5(a-b)). Subsequently, functional experiments showed that the inhibition of NSCLC multiplication, metastasis, and radioresistance caused by circ_0001287 up-regulation could be partially reversed by miR-21 mimics (Figure 5(c-f)); miR-21 inhibitors partially weakened the enhancement of NSCLC multiplication, metastasis, and radioresistance induced by circ_0001287 knockdown (Figure 5(c-f)). Besides, PTEN expression was positively correlated with circ_0001287 expression and negatively associated with miR-21 expression in NSCLC tissues (Supplementary Figure 1). These results indicated that circ_0001287 suppressed NSCLC progression via sponging miR-21 and indirectly up-modulating PTEN expression.

(a) miR-21 mimics and miR-21 inhibitors were transfected into the circ_0001287-H1299 cell model and the si-circ_0001287- A549 cell model, respectively. MiR-21 expression in cells was detected using qRT-PCR. (b) Western blot was used to detect the expression of PTEN protein in NSCLC cells. (c–d) Cell multiplication was detected by CCK-8 method and BrdU experiment. (e) Transwell assay was employed to detect cell migration and invasion of NSCLC cells. (f) Under different doses of radiation, the survival of NSCLC cells was tested using colony formation experiments. The experiments were repeated three times. **#** *P* < 0.05, ** **##** *P* < 0.01, and *** **###** *P* < 0.001.

## Discussion

As a new type of ncRNA to regulate gene expression, circRNA is firstly found in viruses and it is considered to be aberrant splicing by-products [24]. Moreover, circRNA is widely recognized as an essential player in regulating cell structure, tissue homeostasis, and physiological and pathological processes [25,26]. Accumulating studies report that abnormally expressed circRNAs are crucial to the progression of NSCLC and the prognosis of the patients. For instance, circPTPRA impedes the epithelial-mesenchymal transition and metastasis of NSCLC cells [27]; circ_ARHGAP10 promotes the progression of NSCLC by up-modulating GLUT-1 expression, and its overexpression indicates the unfavorable prognosis of NSCLC patients [28]; circ_100395 inhibits the malignant phenotypes of lung cancer cells via modulating TCF21 expression [29]. The present work authenticated that circ_0001287 expression was down-modulated in NSCLC tissues and cells. Additionally, circ_0001287 under-expression was linked to poor differentiation and positive lymph node metastasis of NSCLC patients. Functionally, circ_0001287 inhibited the multiplication, migration, and invasion in NSCLC, and enhanced its radiosensitivity. These results verified that circ_0001287 was a promising biomarker and therapeutic target of NSCLC.

MiRNAs, with 18–24 bases in length, regulate a series of biological processes, including cell survival, multiplication, apoptosis, tumor growth, and metastasis [30]. MiR-21 is considered to be an oncogene in a variety of cancers [31,32]. Importantly, miR-21 enhances cell multiplication, metastasis, and radioresistance, and suppresses apoptosis of NSCLC cells, and it is reported as a novel biomarker for screening early-stage NSCLC [33–36]. Consistent with these previous studies, herein, our research showed that miR-21 promoted the malignant phenotypes of NSCLC cells. Moreover, circRNA can work as competitive endogenous RNA (ceRNA), also called molecular sponge, to negatively regulate the expressions of miRNAs, contributing to tumor progression [37]. For example, in NSCLC, circ_100146 exerts oncogenic function by adsorbing miR-361-3p and miR-615-5p [38]. In this work, bioinformatics analysis implied a possible binding site between circ_0001287 and miR-21. The luciferase reporter experiment, RIP experiment and qRT-PCR confirmed that circ_0001287 could sponge miR-21 and negatively modulate its expression. Subsequently, we demonstrated that miR-21 partially counteracted the inhibiting effects of circ_0001287 on multiplication, migration, invasion, and radioresistance of NSCLC. These data indicated that circ_0001287 could act as a ceRNA for miR-21 in NSCLC to play a tumor-suppressive role.

Loss-of-function mutations or suppression of PTEN drives the development of diverse human cancers, including NSCLC [39,40]. PI3K signaling is one of the most crucial pathways in cancer biology, regulating cell cycle progression, survival, migration, invasion, and metabolism of cancer cells, and PTEN is the main negative regulator of it by dephosphorylating PIP_3_ to PIP_2_ [39,40]. PTEN also regulates chromosome stability, DNA repair, and apoptosis as a protein phosphatase [39,40]. PTEN mutations are frequent in different cancer types; besides, PTEN expression is down-regulated at both transcriptional and post-transcriptional levels. MiR-21 is one of the most crucial miRNAs which regulates PTEN expression [19–23]. MiR-21/PTEN axis contributes to the multiplication, metastasis, chemoresistance, and radioresistance of NSCLC cells [19–23]. In this work, we confirmed that PTEN could be directly regulated by miR-21 and indirectly by circ_0001287 in NSCLC. Our study provides a novel mechanism to explain the dysregulation of PTEN in NSCLC.

## Conclusion

Circ_0001287 expression is down-modulated in NSCLC, and its low expression is associated with low degree of tissue differentiation and positive lymph node metastasis. Circ_0001287 sponges miR-21 to up-regulate PTEN expression and inhibits the multiplication, metastasis, and radioresistance of NSCLC cells.

## Supplementary Material

Supplemental Material

## Data Availability

The data used to support the findings of this study are available from the corresponding author upon request.

## Highlights

- Circ_0001287 is down-regulated in NSCLC tissues and cell lines.
- Circ_0001287 overexpression suppresses the proliferation, migration, invasion, and radioresistance of NSCLC cells.
- Circ_0001287 can adsorb miR-21 and repress its expression, and indirectly up-regulate the expression of PTEN.
