# Supplementary material for: Circular RNA circ_0001287 inhibits the proliferation, metastasis, and radiosensitivity of non-small cell lung cancer cells by sponging microRNA miR-21 and up-regulating phosphatase and tensin homolog expression
Source: Bioengineered. 2021 Jan 20;12(1):414–25. doi: 10.1080/21655979.2021.1872191 (PMC8806200; doi:10.1080/21655979.2021.1872191)
Supplement: Supplemental Material [file KBIE_A_1872191_SM0016.docx]

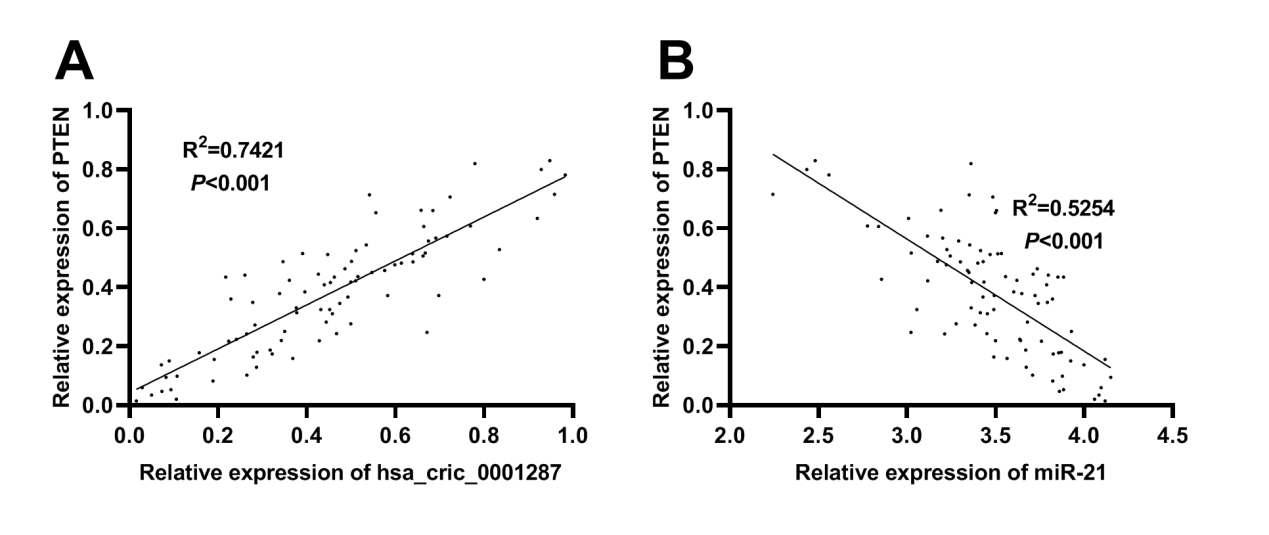


**Supplementary Figure 1**

1. Pearson’s correlation analysis showed that circ_0001287 expression was positively correlated with the expression level of PTEN in NSCLC tissues of the patients.
2. Pearson’s correlation analysis demonstrated that miR-21 expression was negatively correlated with PTEN expression in NSCLC tissues of the patients.
